# Effect of diode laser and Cervitec plus® on dentinal hypersensitivity among chronic periodontitis patients

**DOI:** 10.6026/9732063002001998

**Published:** 2024-12-31

**Authors:** Vidhuta Sareen, Vandana Sarangal, Supreet Kaur, Harleen Kaur Virk, Gianpreet Kaur Matharu, Kaur Guneet Bawa, Sarleen Kaur Gill

**Affiliations:** 1 Department of Periodontology, Sri Guru Ram Das Institute of Dental Sciences and Research, Amritsar, Punjab, India

**Keywords:** Chlorhexidine varnish, chronic periodontitis, dentinal hypersensitivity, diode laser, pain reduction

## Abstract

The efficacy of diode laser and 1% chlorhexidine varnish, Cervitec Plus, on dentinal sensitivity in patients with chronic periodontitis is of interest to dentists. Among the patients who had developed hypersensitivity after scaling and root planing (SRP), sixty were chosen and divided into two big groups. They underwent either the application of the Cervitec Plus varnish or were treated under diode laser treatment. The two primary treatment groups were subdivided to compare the outcome regarding single and multiple applications for each treatment. The findings were that both diode laser treatment and chlorhexidine varnish offered symptomatic relief when combined with SRP. However, patients receiving diode lasers showed a higher reduction in sensitivity scores, primarily upon repeated applications. Thus, although both treatments are effective, diode laser treatment may present better relief for dentine hypersensitivity cases of chronic periodontitis, especially on repeated applications.

## Background:

The causes of dentinal hypersensitivity are exposed dentinal tubules, mainly resulting from loss of protective enamel or gingival recession [1]. The latter is one of the standard features among patients with chronic periodontitis. The dentinal tubules permit external stimuli to penetrate the dentin, stimulate the nerve endings and produce pain [2]. The hydrodynamic theory was widely accepted as an explanation, suggesting that fluid movement within the tubules triggers nerve responses [3]. The management of dentinal hypersensitivity is based on desensitizing agents in the form of fluoride varnishes and lasers designed to lessen the excitement and pain level of the nerve. Its unpredictability and individual variation make treatment difficult [4]. Therefore, it is of interest to increase and improve understanding and a course of management while emphasizing long-term alleviation of affected patients.

## Methodology:

This double-blinded, randomized, open-label, comparative clinical study recruited 60 patients suffering from generalized chronic periodontitis and dentinal hypersensitivity at the Department of Periodontology, Sri Guru Ram Das Institute of Dental Sciences and Research, Amritsar. Ethical approval was taken and all patients gave written informed consent after following the guidelines of the Declaration of Helsinki, (1975 revised 2013).

## Inclusion and exclusion criteria:

At least 18 years of age and having over two hypersensitive teeth. The VAS score of the patients should be above 3. The patient should be systemically healthy, cooperative and willing to sign informed consent. After the treatment, patients were divided into two groups by scaling and root planing. Exclusion criteria include carious lesions, defective restorations, Endo-Perio Lesions, recent desensitizing therapies, or medicines that influence sensitivity assessment.

## Study groups and treatment:

## Group 1 (Cervitec® Plus):

Subgroup A (n=15) was given a single application of chlorhexidine varnish (Cervitec® Plus) immediately following SRP.

Subgroup B (n=15) was given a second application after 14 days.

## Group 2 (diode laser therapy):

Subgroup A (n=15) was given a single application of diode laser following SRP

Subgroup B (n=15) was given a second application of laser after 14 days

All the participants were recorded for periodontal and sensitivity parameters at baseline, 14 days and one-month post-treatmen

## Diagnostic tests:

## Tactile stimulation test:

A dental explorer was rubbed with slight pressure on open surfaces of the dentine with a sensitive response to pain.

## Air blast test:

An air syringe was used, blowing air into sensitive regions.

## VAS score:

The patient scored their sensitivity levels using the help of a visual analog scale

Periodontal Parameters

## The baseline consisted of the following:

## Plaque index (PI):

It was assessed by the measurement of plaque accumulation

## Sulcus bleeding index (SBI):

Blowing on the sulcus was done to analyze bleeding on probing.

## PPD and CAL:

These were evaluated for periodontal health. Procedure and Safety For Cervitec® Plus, topical chlorhexidine varnish was applied immediately post-SRP. Patients were instructed to refrain from rinsing and not drink for 30 minutes following the application. All treatments with diode laser were delivered non-contact with appropriate eye protection.

## Analysis of data:

Statistical analysis SPSS 17.0 was used to analyze the data. The mean values, standard deviation and periodontal parameters' change in values were calculated. All groups were compared using student t-tests, while the variation within each group was examined over time by paired t-tests.

## Results:

The reduction in the Plaque Index for both the Cervitec® Plus and diode laser groups after one month likely results from the initial full-mouth scaling and root planing (SRP), the "gold standard" for periodontal treatment [5]. In the Cervitec® Plus group, chlorhexidine's long-lasting antimicrobial action, due to its substantive and tissue retention, likely contributed to reduced plaque levels by maintaining effective antimicrobial concentrations [6]. In the diode laser group, the observed benefits may stem from the laser's bactericidal and anti-inflammatory effects, which support periodontal pocket healing, as Moritz *et al.* showed [7]. Additionally, the diode lasers' bio-stimulatory effects encourage secondary dentin production and aid in closing dentinal tubules, thereby potentially easing dentin hypersensitivity. Studies by Yilmaz *et al.* [8] highlight the diode laser's potential for antimicrobial action and tissue repair.

Figure 1 (see PDF) denotes highly significant decreases in the Plaque Index in both Group 1 (Cervitec® Plus) and Group 2 (diode laser) at one month (p < 0.001). In Group 1, the Plaque Index decreased from 2.39 ± 0.013 baselines to 1.37 ± 0.047, while in Group 2, it decreased from 2.49 ± 0.158 to 0.69 ± 0.328. The intra-group comparison revealed that the index was lesser in Group 1A and 1B compared to 2A and 2B, where it was measured to decrease from 2.39 ± 0.012 to 1.77 ± 0.014 and from 2.39 ± 0.014 to 0.97 ± 0.090, respectively. Highly significant decreases in the Plaque Index were observed in both Group 1 (Cervitec® Plus) and Group 2 (diode laser) at one month (p < 0.001). In Group 1, the Plaque Index decreased from 2.39 ± 0.013 baselines to 1.37 ± 0.047, while in Group 2, it decreased from 2.49 ± 0.158 to 0.69 ± 0.328. The intra-group comparison revealed that the index was lesser in Group 1A and 1B compared to 2A and 2B, where it was measured to decrease from 2.39 ± 0.012 to 1.77 ± 0.014 and from 2.39 ± 0.014 to 0.97 ± 0.090, respectively. Bleeding index scores also improved in the Cervitec® Plus group, most likely due to reduced plaque and inflammation caused by scaling. Jiang *et al.* [9] proposed that it improved in the laser group due to a diminution of periodontal inflammation and the elimination of pathogens. However, Burns *et al.* [10] found reduced bleeding. Figure 2 (see PDF) denotes a statistically significant decrease in the sulcus bleeding index between the groups, at a value of p < 0.001 after one month. Group 1, who received the Cervitec Plus at baseline, had a sulcus bleeding of 2.40 ± 0.498, while it decreased to 1.70 ± 0.466 in Group 2, who received a diode laser.

## Intra-group results remained quite similar:

Group 1A dropped to 2.46 ± 0.51 and Group 1B to 1.73 ± 0.50. Group 2A and 2B were reduced to 1.74 ± 0.487 and 1.83 ± 0.51, respectively. A statistically significant decrease in the sulcus bleeding index between the groups occurred at a p < 0.001 after one month. Group 1, who received the Cervitec Plus at baseline, had a sulcus bleeding of 2.40 ± 0.498, while it decreased to 1.70 ± 0.466 in Group 2, who received a diode laser.

## Intra-group results remained quite similar:

Group 1A dropped to 2.46 ± 0.51 and Group 1B to 1.73 ± 0.50. Group 2A and 2B were reduced to 1.74 ± 0.487 and 1.83 ± 0.51, respectively. For pocket probing, Cervitec® Plus showed benefits, most probably in combination with chlorhexidine's anti-inflammatory and bactericidal effects combined with SRP, which helps limit dentine permeability and establish long-term relief. The reduction by the diode laser group is through its photothermal effects, which help in tissue coagulation and the decrease in pro-inflammatory cytokines, as laterated by Muhlemann *et al.* [11]. Figure 3 (see PDF) denotes a statistically significant reduction in probing pocket depth was recorded between Group 1 (Cervitec® Plus) and Group 2 (diode laser) at the one-month mark: Group 1; Group 1 baseline mean depth, 5.03 ± 0.76; Group 1. One month, 4.44 ± 0.77; and Group 2: Group 2 baseline means depth, 5.06 ± 0.78; and Group 2 one month, 3.31 ± 0.82. Intergroup comparisons also agreed; Group 1A diminished to 4.50 ± 0.79 and Group 1B to 4.32 ± 0.75. Group 2A and 2B diminished to 4.50 ± 0.79 and 3.06 ± 0.79, respectively. A statistically significant reduction in probing pocket depth was recorded between Group 1 (Cervitec® Plus) and Group 2 (diode laser) at the one-month mark: Group 1; Group 1 baseline mean depth, 5.03 ± 0.76; Group 1. One month, 4.44 ± 0.77; and Group 2: Group 2 baseline means depth, 5.06 ± 0.78; and Group 2 one month, 3.31 ± 0.82. Intergroup comparisons also agreed; Group 1A diminished to 4.50 ± 0.79 and Group 1B to 4.32 ± 0.75. Group 2A and 2B diminished to 4.50 ± 0.79 and 3.06 ± 0.79, respectively. Increases in the Cervitec® Plus group's CAL would be due to the healing of tissues and the formation of new attachments. That can be attributed to Hashim [12].

Improvements in CAL, as noted by Takei *et al.* [13] after diode laser therapy, might be attributed to its ablative action with the potential for inhibiting inflammatory cell infiltration and cell growth in the healing process. Figure 4 (see PDF) denotes that at baseline, the mean CAL values of Group 1 treated with Cervitec® Plus were 5.88 ± 0.74 and those of Group 2 treated with diode laser as 5.92 ± 0.87. A highly significant CAL gain was found in both groups over one month (p < 0.001). By the end of the month, Group 1's CAL improved from 5.88 ± 0.74 to 5.92 ± 0.87. Group 2 improved from 5.92 ± 0.87 to 5.33 ± 0.75. Further intra-group comparisons revealed more improvements in CAL: Group 1A showed improvement from 5.90 ± 0.65 to 5.44 ± 0.66 and Group 2B from 5.93 ± 0.87 to 3.91 ± 0.84 (p < 0.001). At baseline, the mean CAL values of Group 1 treated with Cervitec® Plus were 5.88 ± 0.74 and those of Group 2 treated with diode laser were 5.92 ± 0.87. A highly significant CAL gain was found in both groups over one month (p < 0.001). By the end of the month, Group 1's CAL improved from 5.88 ± 0.74 to 5.92 ± 0.87. Group 2 improved from 5.92 ± 0.87 to 5.33 ± 0.75. Further intra-group comparisons revealed more improvements in CAL: Group 1A showed improvement from 5.90 ± 0.65 to 5.44 ± 0.66 and Group 2B from 5.93 ± 0.87 to 3.91 ± 0.84 (p < 0.001). A VAS with endpoints for no pain to the worst pain possible was used to assess pain since there is empirical evidence regarding this use in the setting of this study. The permeability of dentin was probably decreased by Cervitec® Plus, thereby limiting exposure to dentinal tubules, usually caused by enamel removal and rough brushing [14]. According to Solis *et al.* Pain relief from Diode laser can be attributed to its inhibitory action on the pro-inflammatory neuropeptide bradykinin [15]. Figure 5 (see PDF) denotes that inter-group comparison shows a significant reduction in VAS scores (air-blast stimulus) in both groups over one month (p < 0.001). In Group 1, the mean VAS score decreased from 8.40 ± 0.49 at baseline to 3.76 ± 0.72 on day 14 and further to 1.16 ± 1.05 after one month. Similarly, Group 2's mean score reduced from 8.50 ± 0.50 at baseline to 0.46 ± 0.50 on day 14 and 0.30 ± 0.46 after one month. Intra-group analysis revealed that in Group 1A, the score dropped from 8.33 ± 0.48 at baseline to 4.06 ± 0.70 on day 14 and 1.93 ± 0.88 after one month. Group 1B exhibited a more significant reduction, from 8.46 ± 0.51 at baseline to 3.46 ± 0.63 on day 14 and 0.40 ± 0.50 after one month. Group 2A's mean score declined from 8.53 ± 0.51 at baseline to 3.53 ± 0.51 on day 14 and 0.60 ± 0.50 after one month. Group 2B reduced from 8.46 ± 0.51 at baseline to 3.40 ± 0.50 on day 14 and 0.00 after one month.

A statistically significant difference in VAS scores was observed between the two groups throughout the study period (p < 0.001). In the inter-group comparison, VAS scores dropped significantly in both groups over one month (p < 0.001). In Group 1, the VAS score decreased from a mean of 8.40 ± 0.49 at baseline to 3.76 ± 0.72 on day 14 and even down to 1.16 ± 1.05 at one month. Group 2 decreased its score from 8.50 ± 0.50 at baseline to 0.46 ± 0.50 on day 14 and 0.30 ± 0.46 after one month. The intra-group comparison showed that the VAS score of Group 1A went down from 8.33 ± 0.48 at baseline to 4.06 ± 0.70 on day 14, then to 1.93 ± 0.88 after one month. Group 1B had a more significant diminution of the baseline 8.46 ± 0.51 to 3.46 ± 0.63 on day 14 and then to 0.40 ± 0.50. Scores for Group 2 were demonstrated in Group 2A to decrease from the baseline of 8.53 ± 0.51 on day 14 to 3.53 ± 0.51 and further to 0.60 ± 0.50, while scores for Group 2B were depressed from 8.46 ± 0.51 on day 14 to 3.40 ± 0.50 and then to 0.00 at one month. There is a statistically significant decrease in VAS scores between the two groups for the study period (p < 0.001). Figure 6 (see PDF) denotes that both treatment groups significantly reduced VAS scores over a month with a p-value < 0.001. Group 1 - Cervitec® Plus: The mean score from VAS was decreased by 4.80 ± 0.40 from baseline, with a score of 8.66 ± 0.47 at the beginning to 3.86 ± 0.62 at day 14 and finally to 1.13 ± 1.10 after one month. The second group, which used the diode laser, also showed a decreasing trend where the baseline VAS score went from 8.73 ± 0.44 to 3.60 ± 0.49 at day 14 and finally to 0.23 ± 0.43 one month later. Over a month, both treatment groups significantly showed a reduction in VAS scores with a p-value < 0.001. Group 1 - Cervitec® Plus the mean score from VAS was decreased by 4.80 ± 0.40 from baseline, with a score of 8.66 ± 0.47 at the beginning to 3.86 ± 0.62 at day 14 and finally to 1.13 ± 1.10 after one month. The second group, which used the diode laser, also showed a decreasing trend where the baseline VAS score went from 8.73 ± 0.44 to 3.60 ± 0.49 at day 14 and finally to 0.23 ± 0.43 one month later. The same applied in the intra-group comparison. In Group 1A, the VAS score decreased from 8.66 ± 0.48 to 4.06 ± 0.70 at day 14 and 2.0 ± 0.84 at one month. Group 1B decreased from 8.66 ± 0.48 to 3.66 ± 0.48 to as low as 0.26 ± 0.45 in one month. In group 2, VAS scores for 2A reduced from 8.73 ± 0.45 to 4.06 ± 0.70 by day 14 and then to 0.46 ± 0.51 at one month, while in group 2, B scores reduced from 8.73 ± 0.45 to 3.53 ± 0.51 and at one month became 0.00 ± 0.00. Improvement was statistically significant in all groups (p < 0.001).

## Conclusion:

We show that Diode Laser and Cervitec® Plus treatments significantly reduced dentinal hypersensitivity and improved periodontal parameters in chronic periodontitis patients. While both treatments showed improvements in plaque index, sulcus bleeding index, probing pocket depth and clinical attachment level, the diode laser treatment demonstrated more significant reductions and more excellent pain relief after 30 days. In conclusion, both therapies were effective, but the diode laser showed enhanced results, warranting further studies with larger sample sizes and longer follow-ups to confirm these findings.
